# Feasibility, reproducibility, clinical value of the VExUS score after pediatric cardiac surgery and main differences from adults’ perspective

**DOI:** 10.1007/s00431-026-06999-z

**Published:** 2026-05-08

**Authors:** Daniel Palanca Arias, Marcos Clavero Adell, Aida Lorente López, Ariadna Ayerza Casas, Victoria Estabén Boldova, Irene Gil Hernández, Almudena Alonso Ojembarrena

**Affiliations:** 1 Pediatric Intensive Care Unit, Hospital Universitario Miguel Servet, University of Zaragoza, Zaragoza, Spain; 2 Pediatric Cardiology, Hospital Universitario Miguel Servet, University of Zaragoza, Zaragoza, Spain; 3 Department of Pediatric, Hospital Universitario Miguel Servet, Zaragoza, Spain; 4 Emergency Department, Hospital Clínico Universitario Lozano Blesa, Zaragoza, Spain; 5 Pediatric Intensive Care Unit, Hospital Universitario Miguel Servet, Zaragoza, Spain; 6 Neonatal Intensive Care Unit, Puerta del Mar University Hospital, Cadiz, Spain; 7 Biomedical Research and Innovation Institute of Cadiz (INiBICA), Puerta del Mar University Hospital, Cadiz, Spain

**Keywords:** Cardiac surgical procedures, Doppler, Echocardiography, Hepatic veins, Intensive care units, pediatric, Pediatrics, Portal vein, Vena cava, inferior, Ultrasonography, Venous congestion, Venous excess ultrasound score

## Abstract

The venous excess ultrasound score (VExUS) is a promising method to assess venous congestion in adults, but evidence in children is scarce. This study aimed to evaluate the feasibility, reproducibility, and clinical usefulness of VExUS in pediatric patients. We also explored whether portal venous Doppler (PVD) alone could serve as a faster alternative and assessed the role of inferior vena cava (IVC) measurements. In this prospective single-center study, 35 pediatric patients were enrolled between 2022 and 2024. Associations between clinical variables and VExUS grades at admission (VExUS-0), 24 h (VExUS-24 h), and 48 h (VExUS-48 h), as well as PVD at corresponding time points, were analyzed. The relationship between IVC diameter and VExUS was also evaluated. VExUS demonstrated perfect reproducibility (κ coefficient and intraclass correlation coefficient = 1). Patients with VExUS-0 or VExUS-24 h grades 2–3 had longer aortic cross-clamp times (*p* = 0.03; 0.04) and higher vasoactive–inotropic scores (*p* = 0.01) than those graded 0–1. A higher incidence of acute kidney injury was observed in VExUS-24 h grades 2–3 (*p* = 0.04). Similar associations were found with PVD. Most patients with VExUS grades 2–3 had non-dilated IVCs according to pediatric reference values.

*Conclusion*: VExUS is a feasible, reproducible, and clinically relevant bedside tool for detecting venous congestion in children. Its association with morbidity markers suggests prognostic potential, with optimal performance 24 h after PICU admission. PVD may provide comparable information in less time, while IVC diameter appears unreliable for this purpose.

**Table:** 

**What is Known:** • *VExUS enables bedside assessment of venous congestion and is associated with adverse outcomes in adults, particularly after cardiac surgery.* • *However, pediatric evidence is limited, and its clinical applicability remains uncertain.*	
**What is New:** • *This study shows that VExUS is feasible, reproducible, and associated with morbidity after pediatric cardiac surgery, with the highest prognostic value at 24h.* • *Portal Doppler may provide comparable performance, whereas IVC diameter is is not a reliable marker of venous congestion in this population.*

**Supplementary Information:**

The online version contains supplementary material available at 10.1007/s00431-026-06999-z.

## Introduction

Optimal fluid management is crucial in pediatric intensive care units (PICUs) to prevent venous congestion, which is associated with organ hypoperfusion, subsequent organ dysfunction, and adverse outcomes such as venous hypertension, acute kidney injury (AKI), prolonged mechanical ventilation (MV), and increased morbidity and mortality [1, 2]. Therefore, identifying a reliable and noninvasive method to monitor venous congestion is essential.

The Venous Excess Ultrasound Grading System (VExUS) has been recognized as a valuable tool for assessing venous congestion by evaluating the inferior vena cava (IVC) diameter and Doppler patterns of the hepatic (HVD), portal (PVD), and intrarenal veins (IRVD) [3]. It has been described as an essential bedside “third eye” for intensivists [4] and as a decision-making tool to guide fluid management in critically ill patients [5].

In adult populations, PVD and IRVD are good markers of venous congestion, independently associated with AKI after cardiac surgery [6], and predictive of response to diuretic therapy, showing stronger correlations with central venous pressure (CVP) and NT-proBNP than the overall VExUS score. Further adult studies have confirmed their prognostic relevance in heart failure and after cardiac surgery [7–12].

However, diagnosing and monitoring venous congestion in pediatric patients remains particularly challenging, as there are few references in the pediatric literature. Menéndez-Suso et al. validated the reliability and utility of VExUS for detecting high CVP in critically ill children [13]. Natraj et al. reported the application of VExUS by POCUS-trained pediatric intensivists in children with septic shock [14] and, more recently, in 31 children with right ventricular dysfunction (RVD) following congenital heart surgery, where PVD was the most useful predictor of AKI [15]. Currently, only two recent studies have evaluated the relationship between venous congestion assessed by VExUS and postoperative AKI in pediatric cardiac surgery [16] or its association with tricuspid annular plane systolic excursion (TAPSE) and pulmonary artery systolic pressure (PASP) [17]. However, all these studies have included a reduced number of variables and patients, so more evidence is needed to elucidate the use of VExUS in clinical settings for children.

Furthermore, the ultrasonographic assessment of VExUS in children has several limitations, since IVC diameter correlates with anthropometric variables, underscoring the need for pediatric reference values adjusted for body size [18–21].

The primary objective of this study was to analyze the feasibility, reproducibility, and association between the degree of venous congestion, assessed using VExUS, and prognostic indicators of poor outcome during the first 48 h after PICU admission in children following cardiac surgery. Secondary objectives were to identify presurgical variables associated with higher VExUS grades after admission to PICU, to determine whether PVD is also correlated with these parameters as a faster assessment of venous congestion, and to analyze the relationship between IVC size proposed for pediatric populations in previous references [18, 20] and VExUS grade at different time points during admission.

## Materials and methods

### Study design

A prospective observational study was conducted in children aged 1 month to 15 years admitted to the PICU of a tertiary hospital between December 2022 and December 2024.

### Study population

All patients with congenital heart disease who underwent cardiac surgery via sternotomy with cardiopulmonary bypass (CPB) at Miguel Servet Hospital, Zaragoza, were eligible for inclusion, provided that VExUS assessment could be performed during the study period.

Exclusion criteria included pre-existing AKI, residual or uncorrected cardiac lesions, need for preoperative MV or inotropic support, preoperative cardiac arrest, use of nephrotoxic drugs, diabetes insipidus, chronic pulmonary disease, immunodeficiency, gestational age < 32 weeks, lack of signed informed consent, poor acoustic windows, arrhythmias, aggressive ventilatory settings (positive end-expiratory pressure > 10 cmH_2_O), significant RVD, pulmonary hypertension, or any condition interfering with PVD (e.g., portal vein thrombosis).

### Study objectives

1. Analyze the relationship between VExUS at admission (VExUS-0), 24 h (VExUS-24 h), and 48 h (VExUS-48 h) after surgery and prognostic variables related to the postoperative period of cardiac surgery, grouped into different sections (anthropometric, congenital heart disease and surgery, PICU risk scores, multiorgan congestion profile, and postoperative outcome variables).
2. Analyze whether the same information can be obtained from PVD at admission (PVD-0), 24 h (PVD-24 h), and 48 h (PVD-48 h) after surgery.
3. Determine which presurgical variables are related to VExUS grades 2–3 at admission or 24 h after surgery.
4. Evaluate the intra- and inter-reproducibility of VExUS in children.
5. Compare IVC size at different time points with final VExUS classification for each patient, according to previous pediatric reference studies [18, 20].

### Data collection

VExUS-0, VExUS-24 h, and VExUS-48 h were evaluated in all patients included. Demographic and clinical variables collected at the time of ultrasound assessment included sex, age (months), and weight (kg). Analytical and surgical variables were also recorded: CPB duration, type of cardiac surgery, aortic cross-clamp and ischemia times, and TAPSE as a marker of RVD. ProBNP levels and blood gas analyses were obtained as part of routine care.

Clinical severity and outcome variables were as follows: maximum vasoactive–inotropic score (VIS) [22], Risk Adjustment for Congenital Heart Surgery-1 (RACHS-1) [23] and Aristotle surgical risk category, AKI, fluid balance (mL), Pediatric Risk of Mortality III (PRISM-III) and Pediatric Index of Mortality (PIM) scores [24, 25], length of PICU stay, duration of invasive mechanical ventilation (IMV, hours), maximum lactate (mmol/L), TAPSE (mm), lung ultrasound (LUS) score, and death (all definitions are provided in the Supplementary Material). All patients were followed until hospital discharge or death.

### Ultrasound protocol

Ultrasound examinations were performed using a Canon Aplio a ultrasound system (Canon Medical Systems, Switzerland). A convex probe (Canon PVT-574BT 10C1) with simultaneous ECG tracing (model BR-903F) was used for VExUS assessment, and a linear probe (Canon PLT-1005BT 14L5) for LUS evaluation. For echocardiographic assessment of RVD and TAPSE measurement, a sector probe (Canon PST-28BT 6S1) was used.

All images were obtained by the same senior staff physician (D.P.A.) with over 10 years of experience in PICU and pediatric cardiology. Image analysis was performed blinded to clinical outcomes. Patients were examined in a supine position with the head elevated 0–30°. Mechanically ventilated patients were sedated and, when necessary, pharmacologically paralyzed according to local protocols. Non-sedated patients were examined while calm and cooperative whenever possible.

### Inferior vena cava measurements

The maximum IVC diameter was measured approximately 2 cm below the right atrial junction using a subxiphoid approach with the probe indicator oriented toward the patient’s head. B-mode sagittal images were acquired.

Given the variability of IVC size with age and the lack of pediatric reference Z-scores, venous Doppler evaluation was performed irrespective of IVC diameter. The values were compared with those reported in previous pediatric reference studies [18, 20].

### Portal, hepatic, and intrarenal venous Doppler

HVD, PVD, and IRVD signals were obtained from the right hypochondrium following the methodology described by Beaubien-Souligny et al. [3]. To minimize respiratory variability, recordings were obtained during stable and reproducible flow phases. When multiple patterns were present, the highest-quality tracing—defined by maximal velocity and minimal artifact—was selected for analysis.

Venous Doppler findings were classified according to adult VExUS criteria [3]: normal Doppler = grade 0; mildly abnormal Doppler = grade 1; one severely abnormal Doppler = grade 2; two or more severely abnormal Dopplers = grade 3. Due to the small number of patients with VExUS grade 3, grades 2 and 3 were analyzed together.

### Lung ultrasound

LUS was performed concurrently with routine echocardiography according to institutional postoperative protocols. Examinations were conducted by the same experienced senior physician (D.P.A.). A 12-zone protocol (two anterior, two lateral, and two posterolateral zones per lung) was used, with scoring as follows: 0 = ≤ 2 B-lines; 1 = multiple separated B-lines; 2 = coalescent B-lines; 3 = lung consolidation (complete loss of aeration). The total LUS score (range 0–36) was the sum of all zone scores [26].

### Reproducibility

Intra- and interobserver reproducibility were assessed for all VExUS components, including IVC size, HVD, PVD, and IRVD. Reproducibility analyses were performed in a randomly selected subset of 10 patients, both for image acquisition and interpretation, as explained below: (a) intraobserver variability was evaluated by the primary operator (D.P.A.) through repeated image acquisition in the same patients at different time points, and by repeated measurements on the same stored images; (b) interobserver reproducibility was assessed by a second independent observer (M.C.A.), a physician with more than 7 years of experience in echocardiography and over 3 years of specific experience in VExUS assessment. This observer was blinded to clinical data and to previous measurements. Interobserver agreement was evaluated both by offline analysis of stored images and by independent ultrasound examinations performed in the same patients. Intraclass correlation coefficients (ICC) were used to assess reproducibility of IVC measurements, using a two-way random-effects model for absolute agreement and consistency. Doppler-derived variables, including VExUS grading, were analyzed using weighted Cohen’s kappa coefficient (κ).

### Statistical analysis

Continuous variables were described as medians and interquartile ranges (IQRs), and categorical variables as absolute numbers and percentages. Differences among VExUS categories were analyzed using the Kruskal–Wallis test for quantitative variables and the chi-square or Fisher’s exact test for qualitative variables. Trends for quantitative variables were assessed using the nonparametric Jonckheere–Terpstra test.

Presurgical variables related to VExUS grades 2–3 were included in a multivariate analysis with logistic regression. The odds ratio with 95% confidence interval for each variable was reported.

According to previous publications, the standard deviation of PICU length of stay after pediatric heart surgery is 1.5 days [27, 28]. We estimated a reduction in PICU stay of 2 days in patients with VExUS grades 0–1 at admission compared with those with VExUS grades 2–3. Assuming that the number of patients with VExUS 0–1 is three times that of those with VExUS 2–3, a sample size of 32 patients was calculated to provide 90% power with an alpha error of 5%.

A *p* value < 0.05 was considered statistically significant. Statistical analyses were performed using STATA v18.0 (StataCorp, College Station, TX, USA).

### Ethics

Informed consent was obtained from the parents or legal guardians of all patients after receiving detailed information about the study. Data were fully anonymized, and all identifying information was removed. Each participant was assigned a unique study code known only to the investigators.

The study was approved by the Research Ethics Committee of the Autonomous Community of Aragon (CEICA) (IRB: C.I. PI22/538). All procedures were conducted in accordance with the ethical principles outlined in the Declaration of Helsinki (1964 and subsequent revisions).

## Results

### Population

**Fig. 1:**
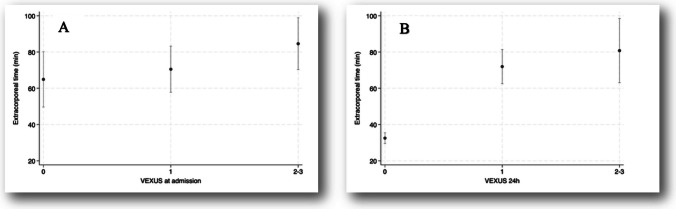
Relationship between VExUS at admission (**A**) and at 24 h (**B**), with extracorporeal time. **A** Comparison between groups, *p* = 0.29 (Kruskal–Wallis test); tendency between groups, *p* = 0.13 (Jonckheere–Terpstra test); **B** Comparison between groups, *p* = 0.05 (Kruskal–Wallis test); tendency between groups, *p* = 0.04 (Jonckheere–Terpstra test)

Thirty-five patients were analyzed during the study period (Table 1S). The flow diagram of patient inclusion is shown in Fig. 1S.

Intra- and inter-reproducibility for image acquisition and image interpretation was analyzed separately in 10 additional patients, encompassing 48 VExUS examinations (grades 0–3). Cohen’s kappa for HVD, PVD, and IRVD was 1.0 (95% CI 1.0–1.0) for both intra- and inter-reproducibility in image acquisition and image interpretation. Intraclass correlation coefficients for IVC measurements also showed perfect reproducibility (Table 2S).

### Univariate analysis between VExUS, PVD, and clinical variables

**Fig. 2:**
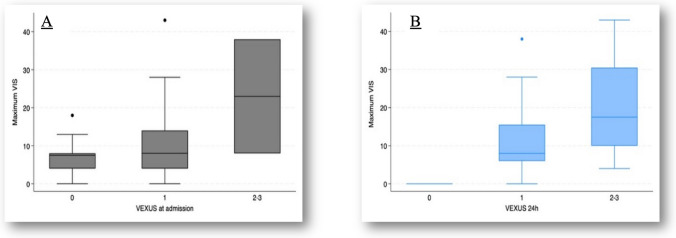
Relationship between VExUS and maximum VIS at admission (**A**) and at 24 h (**B**). **A** Comparison between groups, *p* = 0.01 (Kruskal–Wallis test); tendency between groups, *p* = 0.01 (Jonckheere–Terpstra test); **B** Comparison between groups, *p* = 0.01 (Kruskal–Wallis test), tendency between groups, *p* = 0.004 (Jonckheere–Terpstra test); VIS: vasoactive–inotropic score

**Fig. 3:**
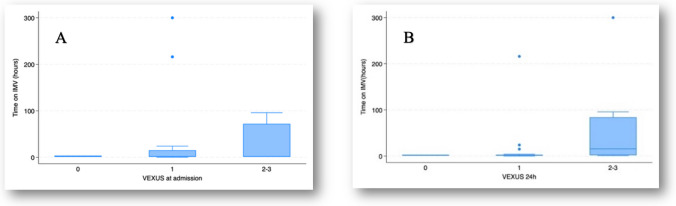
Relationship between VExUS at admission (**A**) and 24 h (**B**), with time on invasive mechanical ventilation. **A** Comparison between groups, *p* = 0.88 (Kruskal–Wallis test); tendency between groups, *p* = 0.88 (Jonckheere–Terpstra test); **B** Comparison between groups, *p* = 0.11 (Kruskal–Wallis test); tendency between groups, *p* = 0.04 (Jonckheere–Terpstra test)

**Fig. 4:**
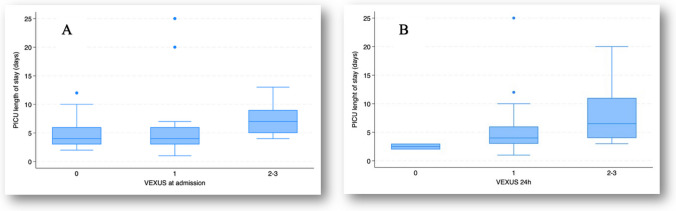
Relationship between VExUS at admission (**A**) and 24 h (**B**), and length of PICU days. PICU: pediatric intensive care unit. **A** Comparison between groups, *p* = 0.10; tendency between groups, *p* = 0.07 (Jonckheere–Terpstra test); **B** Comparison between groups, *p* = 0.09; tendency between groups, *p* = 0.04 (Jonckheere–Terpstra test)

We analyzed the relationship between different clinical variables and VExUS-0 or VExUS-24 h (Table 1) and between these variables and PVD-0 or PVD-24 h (Table 2). The relationships between VExUS-48 h and maximum VIS and hospital length of stay are presented in the Supplementary Material (Figs. 2S and 3S), as well as the associations between VExUS at admission and 48 h after surgery and TAPSE (Fig. 4S).

**Table 1:** Clinical and surgical variables, including risk scores, fluid status, and postoperative outcomes, according to VExUS grade

		At admission				After 24 h			
		VExUS 0 (*n* = 10)	VExUS 1 (*n* = 18)	VExUS 2–3 (*n* = 7)	*p* value	VExUS 0 (*n* = 2)	VExUS 1 (*n* = 25)	VExUS 2–3 (*n* = 8)	*p* value
Anthropometry	Male	5 (50%)	12 (67%)	2 (29%)	0.22	1 (50%)	12 (48%)	6 (75%)	0.41
	Age (months)	20 (6–19)	22 (12–72)	90 (14–180)	0.37	77 (69–84)	21 (12–72)	48 (4–138)	0.65
	Weight (kg)	11.5 (5.9–20)	11 (7.5–22)	8 (6–25)	0.40	20 (11–20)	11 (8–22)	17.4 (6–29)	0.67
Congenital heart disease and surgery	Aristotle risk score	6 (3–6)	6 (3–6)	6 (6–6)	0.46	3 (3–3)	6 (6–6)	6 (6–7)	0.06
	RACHS-1	2 (1–2)	2 (1–2)	2 (2–2)	0.13	1 (1–1)	2 (1–2)	2 (2–2.5)	0.03
	Preoperative ProBNP (pg/ml)	421 (113–696)	342 (156–854)	1673 (170–3636)	0.42	245 (12–478)	279 (165–823)	3161 (472–4419)	0.24
	ACC time (min)	41 (21–59)	44 (35–63)	66 (61–81)	0.03	11 (0–21)	50 (35–70)	55 (41–75)	0.04
PICU risk score scales	PRISM-III	7 (5–10)	6 (5–10)	12 (5–15)	0.15	8 (5–11)	7 (5–10)	7 (3–12)	0.63
	PIM2	1.5 (0.9–1.8)	0.83 (0.54–1.1)	0.72 (0.54–0.98)	0.07	0.62 (0.58–0.65)	0.89 (0.54–1.42)	0.87 (0.71–1.3)	0.55
Multiorgan congestion profile	AKI	1 (6%)	5 (28%)	2 (29%)	0.66	0 (0%)	4 (16%)	5 (63%)	0.04
	Fluid balance after 24 h (ml)	74 (− 63; + 220)	147 (− 104; + 306)	− 90 (− 951; + 159)	0.27	+ 156 (− 20; + 337)	− 135 (− 442; − 24)	− 10 (− 431; + 194)	0.85
	LUS after 24 h	8 (6–10)	9 (6–13)	15 (4–19)	0.82	7 (6–7)	9 (6–12)	15 (9–19)	0.14
Postoperative outcomes	TAPSE after 24 h (mm)	11 (9.6–14)	8.5 (7.4–10.4)	7.1 (5.5–9)	0.03	22 (14–30)	9 (8–10)	7 (6–18)	0.08
	PICU stay (days)	4 (3–6)	4 (3–6)	7 (5–9)	0.07	3 (2–3)	4 (3–6)	7 (4–11)	0.09
	IMV (hours)	3 (1–3)	2 (1–15)	2 (1–72)	0.88	2 (1–3)	2 (1–3)	16 (2–84)	0.11
	Maximum lactate (mmol/L)	1.9 (1.7–2.3)	1.7 (1.3–2.6)	2.3 (1.7–6)	0.39	2.3 (2.3–2.3)	1.7 (1.3–2.1)	2.8 (2.1–3.9)	0.046
	Maximum VIS	7.5 (4–8)	8 (4–14)	23 (8–38)	0.01	0 (0–8)	8 (6–16)	18 (10–31)	0.01
	Death	0 (0%)	1 (6%)	0 (0%)	0.62	0 (0%)	0 (0%)	1 (12.5%)	0.29

Categorical variables are presented as absolute number and percentage. Continuous variables are expressed as median (interquartile range). Chi-square test is used for qualitative variables, and ANOVA/Kruskal–Wallis test for quantitative variables (as appropriate). *ACC*, aortic cross-clamp; *LUS*, lung ultrasound aeration score (dimensionless variable); *PRISM-III*, Pediatric Risk of Mortality III; *PIM*, Pediatric Index of Mortality; *TAPSE*, tricuspid annular plane systolic excursion; *PICU*, pediatric intensive care unit; *IMV*, invasive mechanical ventilation; *VIS*, vasoactive–inotropic score

**Table 2:** Clinical and surgical variables, including risk scores, fluid status, and postoperative outcomes, according to portal venous Doppler grade

		At admission				After 24 h			
		PVD 0 (*n* = 15)	PVD 1 (*n* = 16)	PVD 2–3 (*n* = 4)	*p* value	PVD 0 (*n* = 14)	PVD 1 (*n* = 13)	PVD 2–3 (*n* = 8)	*p* value
Anthropometry	Male	8 (53%)	9 (56%)	2 (50%)	0.97	9 (64%)	4 (31%)	6 (75%)	0.09
	Age (months)	25 (7–84)	24 (10–114)	20 (10–102)	0.99	16 (8–72)	25 (14–96)	48 (14–138)	0.76
	Weight (kg)	14 (7–22)	12.2 (7.3–27.2)	10.1 (6.7–25.4)	0.98	12 (7–20)	11 (8–25)	17 (8–29)	0.69
Congenital heart disease and surgery	Aristotle risk score	6 (3–6)	6 (4.5–6)	6 (6–7)	0.39	6 (3–6)	6 (6–6)	6 (5–7)	0.57
	RACHS-1	2 (1–2)	2 (2–2)	2 (2–3)	0.43	2 (1–2)	2 (2–2)	2 (2–3)	0.35
	Preoperative ProBNP (pg/ml)	279.2 (133.8–569.2)	306.5 (169–639)	3161 (1716–4869)	0.06	491 (134–1716)	185 (165–569)	472 (208–3161)	0.63
	ACC time (min)	40 (21–66)	46.5 (37–72)	70 (63–82)	0.04	49 (23–75)	42 (35–63)	55 (39–75)	0.65
PICU risk score scales	PRISM-III	7 (5–10)	6 (5–11)	12 (10–14)	0.14	8 (6–11)	5 (5–7)	10 (4–14)	0.23
	PIM2	0.89 (0.58–1.72)	0.8 (0.55–1.13)	0.86 (0.78–1)	0.71	0.91 (0.58–1.42)	0.89 (0.56–1.41)	0.8 (0.6–1.01)	0.91
Multiorgan congestion profile	AKI	2 (13%)	4 (25%)	3 (75%)	0.08	4 (28%)	0 (0%)	5 (63%)	0.01
	Fluid balance after 24 h (ml)	90 (− 134.1; + 333.1)	54.8 (− 206; + 230)	137 (− 672; + 278)	0.87	29 (− 104; + 305)	121 (− 108; + 273)	108 (− 194; + 176)	0.97
	LUS score after 24 h	6 (4–10)	11 (7–13)	16 (11–18)	0.03	8 (6–19)	7 (4–10)	13 (9–16)	0.16
Postoperative outcomes	TAPSE after 24 h (mm)	10 (9.1–14)	8.5 (6.5–10.4)	6.6 (5.5–7.5)	< 0.01	9.6 (8.5–14)	9 (7.4–11)	7.1 (5.1–18)	0.31
	PICU stay (days)	4 (3–6)	4.5 (3–6)	11 (8–19)	0.02	4 (3–7)	4 (3–6)	7 (4–11)	0.30
	IMV (hours)	2 (1–3)	3 (1–20)	84 (37–156)	0.06	3 (1–3)	1 (1–3)	16 (2–84)	0.10
	Maximum lactate (mmol/l)	1.9 (1.7–2.3)	1.7 (1.2–2.9)	2.4 (1.7–4.5)	0.61	1.8 (1.3–2.3)	2.1 (1.3–4.8)	2.8 (1.7–3.9)	0.18
	Maximum VIS	7.5 (4–8)	8 (7–17)	22 (20–31)	0.02	8 (4–8)	8 (6–13)	22 (10–33)	0.04
	Death	0 (0%)	1 (6%)	0 (0%)	0.54	0 (0%)	0 (0%)	1 (13%)	0.18

Categorical variables are presented as absolute number and percentage. Continuous variables are expressed as median (interquartile range). Chi-square test is used for qualitative variables, and ANOVA/Kruskal–Wallis test for quantitative variables (as appropriate). *ACC*, aortic cross-clamp; *LUS*, lung ultrasound aeration score (dimensionless variable); *PRISM-III*, Pediatric Risk of Mortality III; *PIM*, Pediatric Index of Mortality; *TAPSE*, tricuspid annular plane systolic excursion; *PICU*, pediatric intensive care unit; *IMV*, invasive mechanical ventilation; *VIS*, vasoactive–inotropic score

Figures 1, 2, 3 and 4 illustrate the relationships between VExUS-24 h and extracorporeal circulation time, maximum VIS, IMV duration, and PICU length of stay. These findings suggest that higher degrees of congestion assessed by VExUS-24 h are associated with more complex perioperative parameters and less favorable clinical outcomes.

### Variables related to VExUS grades 2–3 at 24 h

Presurgical variables and variables assessed at admission in relation to VExUS-0 grades 2–3 and VExUS-24 h grades 2–3 are presented in Supplementary Tables 3S and 4S, respectively, according to the multivariate analysis. No significant associations were identified with any of the evaluated variables.

### Analysis of IVC size

In the analysis of IVC size at admission and at 24 h after surgery, 50–75% of patients classified as VExUS grades 2–3 at either time point had non-dilated IVC values according to previous pediatric reference studies [18, 20] (Table 5S).

## Discussion

This study demonstrates that both VExUS-24 h and PVD-24 h in children after cardiac surgery are associated with variables related to clinical severity (maximum VIS, aortic cross-clamp time, and AKI), in line with findings from adult cohorts. VExUS-24 h was also associated with more prolonged mechanical ventilation and longer PICU stays, supporting its potential prognostic value in this setting.

We also evaluated the optimal timing for VExUS assessment after surgery. Our results suggest that 24 h after PICU admission is the most informative time point for detecting clinically relevant venous congestion. At admission, it may be too early to identify significant postoperative venous congestion, as the effects of surgery and perioperative management may evolve gradually. In contrast, at 48 h, many patients have improved clinically and have already received targeted treatments such as inotropes or diuretics, which may modify venous Doppler patterns and mask the initial severity of congestion.

Although VExUS is not a technically complex examination [29], it requires adequate training and experience to ensure reliable measurements. On the other hand, in pediatric patients, the choice of a convex probe allows for good image acquisition by adjusting the scale for IRVD. Another barrier may be the occasional lack of a simultaneous ECG tracing in some PICU ultrasound equipment, which is important for interpreting HVD. Previous authors have proposed simplifying the assessment by focusing on a single component when feasible [7, 15, 17]. In our cohort, PVD-24 h showed similar diagnostic performance to VExUS-24 h, which may reduce the time required for bedside assessment and facilitate wider implementation, even in PICUs with more limited ultrasound expertise.

Beaubien-Souligny et al. [6] advocated early detection and correction of abnormal venous flows (PVD and IRVD) after cardiac surgery. In line with this, our findings support the use of VExUS—or at least PVD alone—as part of the postoperative assessment of pediatric patients after cardiac surgery.

Natraj et al. described VExUS as a logical extension of POCUS, emphasizing that pediatric intensivists with appropriate training can reliably assess venous flows to detect venous congestion, which may contribute to tissue hypoperfusion [15]. They found a significant association between severe congestion (VExUS grade 3) at 24 h and prolonged CPB, as well as improvement in RVD and VExUS with fluid removal. In our study, we observed longer CPB times, longer IMV duration, and higher maximum VIS in patients with VExUS-24 h grades 2–3, as well as lower TAPSE values at admission for both VExUS-0 and PVD-0.

Although only a few studies have evaluated VExUS in pediatric populations, just two have focused on the postoperative period of cardiac surgery. The retrospective study by Cao et al. [16], which included a larger number of patients after cardiac surgery, did not find significant differences in variables associated with postoperative failure (PICU stay, VIS, surgical times, or IMV duration) according to VExUS-24 h. However, that study performed only a single VExUS measurement, without monitoring dynamic changes over time, and did not report the type of cardiac surgery, surgical risk scales, or VIS, limiting comparisons with other studies. In our prospective study, we identified additional variables that may influence prognosis, such as preoperative ProBNP and mortality risk scores (RACHS-1, PIM2), which could reach statistical significance in larger cohorts.

In another recent study of pediatric patients following cardiac surgery, with a sample size similar to ours (43 children) and daily ultrasound evaluations during the first three postoperative days, Siuba et al. [17] observed an association between increasing VExUS scores and worsening of the expected TAPSE-to-PASP ratio. In our study, TAPSE was related to VExUS-0 and PVD-0 as well. It would have been of interest to have preoperative TAPSE data and follow-up measurements after PICU discharge, as well as additional parameters of RVD such as the tricuspid S′ wave or TAPSE/PASP.

We also analyzed IVC size at admission and 24 h after surgery and found that at least half of the patients classified as VExUS grades 2–3 at either time point had a non-dilated IVC according to pediatric reference values [18, 20]. In the study by Natraj et al. [15], IVC dilatation was defined using IVC-to-aorta ratio cut-offs. In children, clinical decision-making should probably not rely on IVC size alone, as significant congestion often occurs with a non-dilated IVC. Pediatric IVC may be more compliant than in adults and may not dilate even when venous congestion is present and affecting organ perfusion. Furthermore, all IVC measurements in our study were obtained in the longitudinal axis, which may limit comparison with other series. Further research is needed before specific recommendations on IVC size can be provided.

### Strengths

Although the sample size was relatively small (35 patients), data were collected prospectively, and VExUS was assessed with simultaneous electrocardiographic recording and LUS in all patients by the same trained operator, minimizing interobserver variability and improving the accuracy of the examinations. In addition, simpler assessments such as PVD may be easier to implement in routine clinical practice and still provide relevant prognostic information, particularly regarding PICU length of stay and inotropic requirements.

### Limitations

This study has several limitations. First, it was a single-center study with a limited number of pediatric cardiac surgeries, which may reduce generalizability to other PICUs with different case mixes. Some comparisons may have been underpowered due to the small number of patients in each VExUS category, and future studies with larger samples are warranted.

Second, we did not systematically record additional echocardiographic parameters of RVD (e.g., tricuspid S′, FAC, RV strain) or signs of pulmonary hypertension that might be related to higher VExUS scores or limit the assessment of the pathophysiology of congestion. This could represent an important area for future research.

Third, our cohort included patients with relatively short hospital stays and low mortality rates, which may limit the extrapolation of our findings to units caring for more complex patients. In such settings, VExUS at admission might be more closely related to postoperative complications and long-term outcomes.

## Conclusions

This study identified a relationship between venous congestion, measured by VExUS and/or PVD, and several indicators of severity after pediatric cardiac surgery. The most informative time point to detect associations with clinical outcomes was 24 h after PICU admission. At this time, complete VExUS and isolated PVD showed similar diagnostic performance. Most patients with VExUS grades 2–3 had normal IVC size according to previous pediatric studies, suggesting that IVC measurements alone are not reliable markers of venous congestion in this population.

## Supplementary Information

Below is the link to the electronic supplementary material.

ESM 1 (DOCX.28.3 KB)

ESM 2 (JPG.861 KB)

ESM 3 (JPG.456 KB)

ESM 4 (JPG.386 KB)

ESM 5 (JPG.394 KB)

## Data Availability

No datasets were generated or analysed during the current study.

## Abbreviations

ACC: Aortic cross-clamp time
AKI: Acute kidney injury
ASD: Atrial septal defect
AVSD: Atrioventricular septal defect
CPB: Cardiopulmonary bypass
CVP: Central venous pressure
CEICA: Ethics Committee of the Autonomous Community of Aragon
HVD: Hepatic venous Doppler
IVC: Inferior vena cava
ICCc: Intraclass correlation coefficient
IQRs: Interquartile ranges
IRVD: Intrarenal venous Doppler
LUS: Lung ultrasound score
MV: Mechanical ventilation
PIM: Pediatric Index of Mortality
PICUs: Pediatric intensive care units
PRISM-III: Pediatric Risk of Mortality III
PVD: Portal venous Doppler
PASP: Pulmonary artery systolic pressure
RVD: Right ventricular dysfunction
RACHS-1: Risk Adjustment for Congenital Heart Surgery-1
TAPSE: Tricuspid annular plane systolic excursion
VIS: Vasoactive–inotropic score
VExUS: Venous excess ultrasound score
VSD: Ventricular septal defect
