# Supplementary material for: Feasibility, reproducibility, clinical value of the VExUS score after pediatric cardiac surgery and main differences from adults’ perspective
Source: Eur J Pediatr. 2026 May 8;185(6):371. doi: 10.1007/s00431-026-06999-z (PMC13156094; doi:10.1007/s00431-026-06999-z)
Supplement: Supplementary file 1 — (DOCX.28.3 KB) [file 431_2026_6999_MOESM1_ESM.docx]

**Supplementary material**

1. Definitions and scoring systems used
2. Flowchart of patient selection **(Fig.1S)**
3. Relationship between VExUS-48h and maximum VIS **(Fig.2S)**
4. Relationship between VExUS-48h and length of PICU stay. **(Fig.3S)**
5. Relationship between VExUS at admission and 48 hours and TAPSE **(Fig.4S)**
6. Baseline characteristics of the study population **(Table 1S)**
7. Reproducibility of IVC measurements. **(Table 2S)**
8. **Preoperative variables and variables** related to VExUS grades 2-3 at admission and 24 hours. (**Table 3S and 4S).**
9. Size of the IVC according to previous reference pediatric studies (18, 20) at admission and 24 hours after admission, according to their VExUS. (**Table 5S)**

**1. Definitions and scoring systems used.**

1.1 The vasoactive–inotropic score (VIS) was calculated as:

VIS = (dopamine + dobutamine) × 1 + (milrinone × 10) + (adrenaline + noradrenaline + isoproterenol) × 100 (26).

1.2. Congenital heart diseases were classified according to the international **Adjustment for Congenital Heart Surgery-1 (**RACHS-1) and Aristotle surgical risk category (27,28).

1.3. Mortality risk was assessed using **Pediatric Risk of Mortality III (PRISM-III) and** Pediatric Index of Mortality 2 (PIM2) scores, within the first 24 hours of PICU admission (29).

1.4. Acute kidney injury (AKI) was defined as an increase in serum creatinine ≥50% from baseline within 48–72 hours postoperatively (30). Baseline creatinine was defined as the most recent preoperative value within 72 hours before surgery. AKI severity was staged using the modified pediatric RIFLE (pRIFLE) or AKI Network (AKIN) criteria (Akcan-Arikan; Mehta).

1.5. Fluid balance was calculated as the net difference between total fluids input (intravenous and enteral) and output (urine, stool, and pleural/pericardial drainage) over each 24-hour period.

**Supplementary figures**

**Fig. 1S.** Study flowchart of patient selection

**Fig. 2S.** Relationship between VEXUS at 48h with máximum VIS.

**Fig. 3S.** Relationship between VEXUS at 48h and hospital length of stay

**Fig. 4S.** Relationship between VEXUS at admission (4SA) and 48 hours (4SB) and TAPSE.

**Supplementary tables.**

**Table 1S.** Baseline characteristics of the study population.

**Table 2S.** Reproducibility of IVC measurements.

**Table 3S.** Variables related to VExUS 2-3 at admission.

**Table 4S.** Variables related to VExUS 2-3 at 24 hours.

**Table 5S.** Size of the inferior vena cava according to previous reference pediatric studies (18, 20) at admission and 24 hours after admission, according to their VExUS.

**Table 1S.** Baseline characteristics of the study population.

Total number and percentage for qualitative variables. Mean and standard deviation for parametric quantitative variables. Median and interquartile range for non-parametric quantitative variables.

| Male | | 19 (54%) |
| --- | --- | --- |
| Age (months) | | 24 (8 – 96) |
| Weight (kg) | | 12.7 (7 – 25) |
| Length (cm) | | 89 (67 – 129) |
| Aristotle risk score | | 6 (6 – 6) |
| RACHS-1 | |  |
|  | Risk 2 | 10 (29%) |
|  | Risk 3 | 23 (66) |
|  | Risk 4 | 2 (5%) |
| PRISM-III score at admission | | 8.06 (0.72) |
| PIM2 score | | 0.89 (0.57 – 1.41 |
| Type of heart disease | |  |
|  | VSD | 19 (54%) |
|  | ASD | 9 (26%) |
|  | Mitral regurgitation | 6 (17%) |
|  | AVSD | 1 (3%) |
| On heart failure treatment | | 13 (63%) |
| CPB time (min) | | 0 70 (55 – 90) |
| ACC time (min) | | 48 (35 – 70) |
| Preoperative ProBNP (pg/ml) | | 384 (161 – 1230) |
| ProBNP at admission (pg/ml) | | 403 (148 – 3265) |
| ProBNP after 24h (pg/ml) | | 2460 (1018 – 4850) |
| FiO2 at admission (%) | | 35 (25 – 40) |
| Preoperative creatinine (mg/dl) | | 0.34 (0.26 – 0.44) |
| TAPSE at admission (mm) | | 9 (7.5 – 12.5) |

ACC: Aortic cross-clamp time; ASD: atrial septal sefect; AVSD: atrioventricular septal defect; CPB: Cardiopulmonary bypass time; **PIM:** Pediatric Index of Mortality; PRISM-III: **Pediatric Risk of Mortality III;** RACHS-1: **Risk Adjustment for Congenital Heart Surgery-1**; TAPSE: Tricuspid annular plane systolic excursion; VSD: ventricular septal defect.

**Table 2S.** Reproducibility of IVC measurements.

|  | ICCa | ICCc |
| --- | --- | --- |
| Intraobserver interpretation | 1 (0.99-1) | 1 (0.99-1) |
| Intraobserver acquisition | 1 (0.99-1) | 1 (0.99-1) |
| Interobserver interpretation | 0.99 (0.98-1) | 0.99 (0.98-1) |
| Interobserver acquisition | 0.99 (0.98-1) | 0.99 (0.98-1) |

ICCa: absolute intraclass correlation coefficient; ICCc: consistency intraclass correlation coefficient; IVC: inferior vena cava.

**Table 3S. Preoperative variables and variables** related to VExUS-0 **grades** 2-3 at admission.

|  | **OR [CI 95%]** | **p** | |
| --- | --- | --- | --- |
| Age (months) | 1.01 (0.98-1.03) | 0.51 | |
| Male sex | 8.13 (0.40-164) | 0.17 | |
| Pulmonary hypertension | 4.27 (0.23-80.73) | 0.33 | |
| Pre-surgery proBNP (pg/ml) | 1.00 (0.99-1.00) | 0.39 | |
| Global time of surgery (min) | 0.71 (0.42-1.20) | 0.21 | |
| Time of aortic cross-clamp (min) | 1.49 (0.82-2.73) | 0.19 | |
|  | | |  |

**Table 4S.** **Preoperative variables and variables** related to **VExUS-24h grades 2–3** at 24 hours.

|  | **OR [CI 95%]** | **p** |
| --- | --- | --- |
| Age (months) | 1.00 (0.98-1.03) | 0.34 |
| Male sex | 0.01 (0.0001-7.12) | 0.12 |
| Pulmonary hypertension | 713 (0.09-5584149) | 0.15 |
| Pre-surgery proBNP (pg/ml) | 1.00 (0.99-1.01) | 0.08 |
| Global time of surgery (min) | 1.12 (0.93-1.36) | 0.24 |
| Time of aortic cross-clamp (min) | 0.76 (0.54-1.08) | 0.12 |

**Table 5S.** Size of the inferior vena cava according to previous reference pediatric studies (18, 20) at admission and 24 hours after admission, according to their VExUS.

| **AT ADMISSION** | | | |
| --- | --- | --- | --- |
|  | **VExUS 0** | **VExUS 1** | **VExUS 2-3** |
| **KATHURIA (18)** | | | |
| Normal IVC | 10 (100%) | 17 (94%) | 5 (71%) |
| Dilated IVC | 0 (0%) | 1 (6%) | 2 (29%) |
| **HOROZ (20)** | | | |
| Normal IVC | 6 (60%) | 12 (67%) | 4 (57%) |
| Dilated IVC | 4 (40%) | 6 (33%) | 3 (43%) |
| **24 HOURS AFTER ADMISSION** | | | |
| **KATHURIA(18)** | | | |
| Normal IVC | 2 (100%) | 22 (88%) | 6 (75%) |
| Dilated IVC | 0 (0%) | 3 (12%) | 2 (25%) |
| **HOROZ (20)** | | | |
| Normal IVC | 2 (100%) | 16 (64%) | 4 (50%) |
| Dilated IVC | 0 (0%) | 9 (36%) | 4 (50%) |

*IVC: inferior vena cava.*
