# Supplementary figures and images for: Feasibility, reproducibility, clinical value of the VExUS score after pediatric cardiac surgery and main differences from adults’ perspective
Source: Eur J Pediatr. 2026 May 8;185(6):371. doi: 10.1007/s00431-026-06999-z (PMC13156094; doi:10.1007/s00431-026-06999-z)

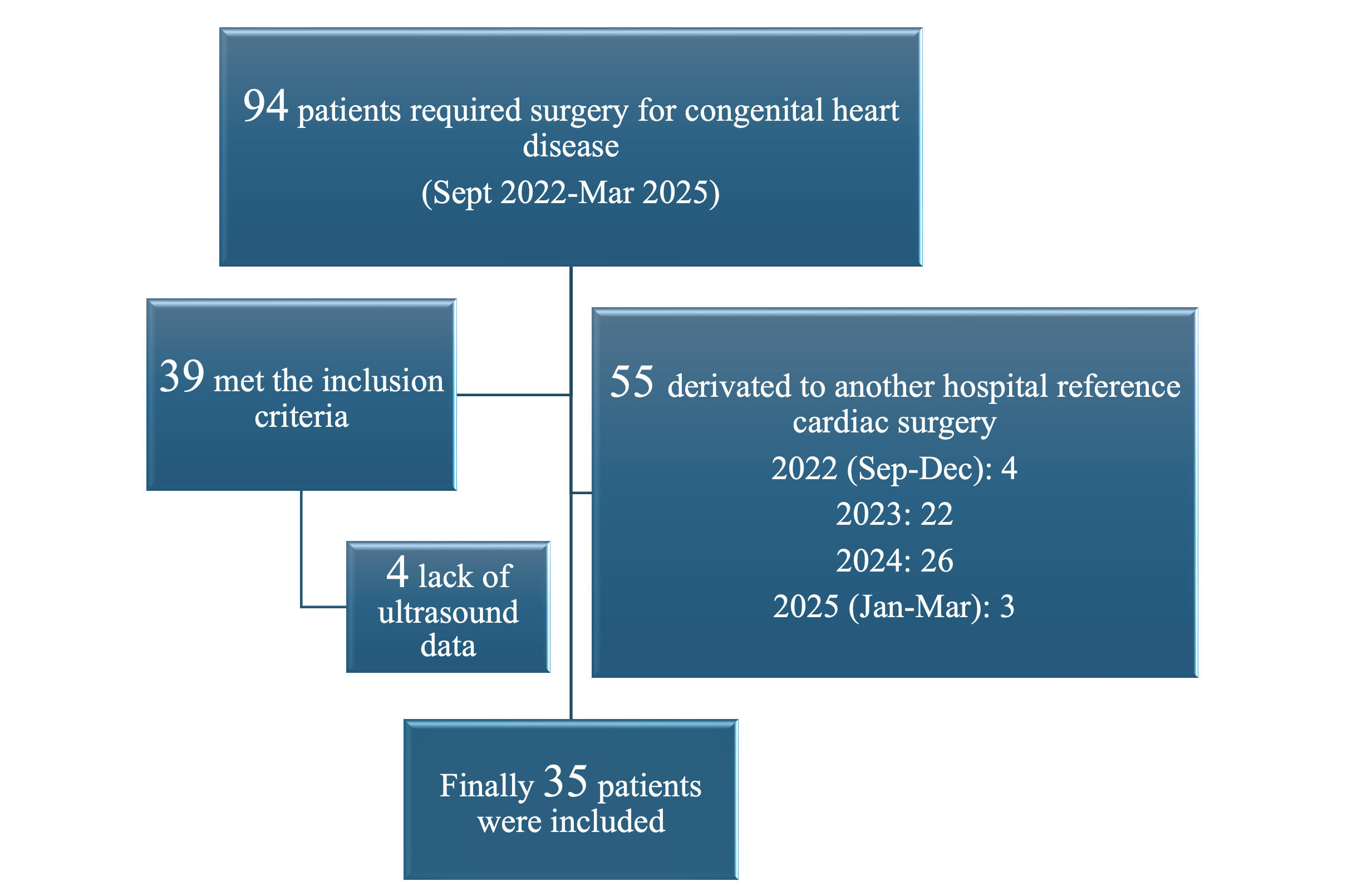

Supplement: Supplementary file 2 — (JPG.861 KB) [file 431_2026_6999_MOESM2_ESM.jpg]

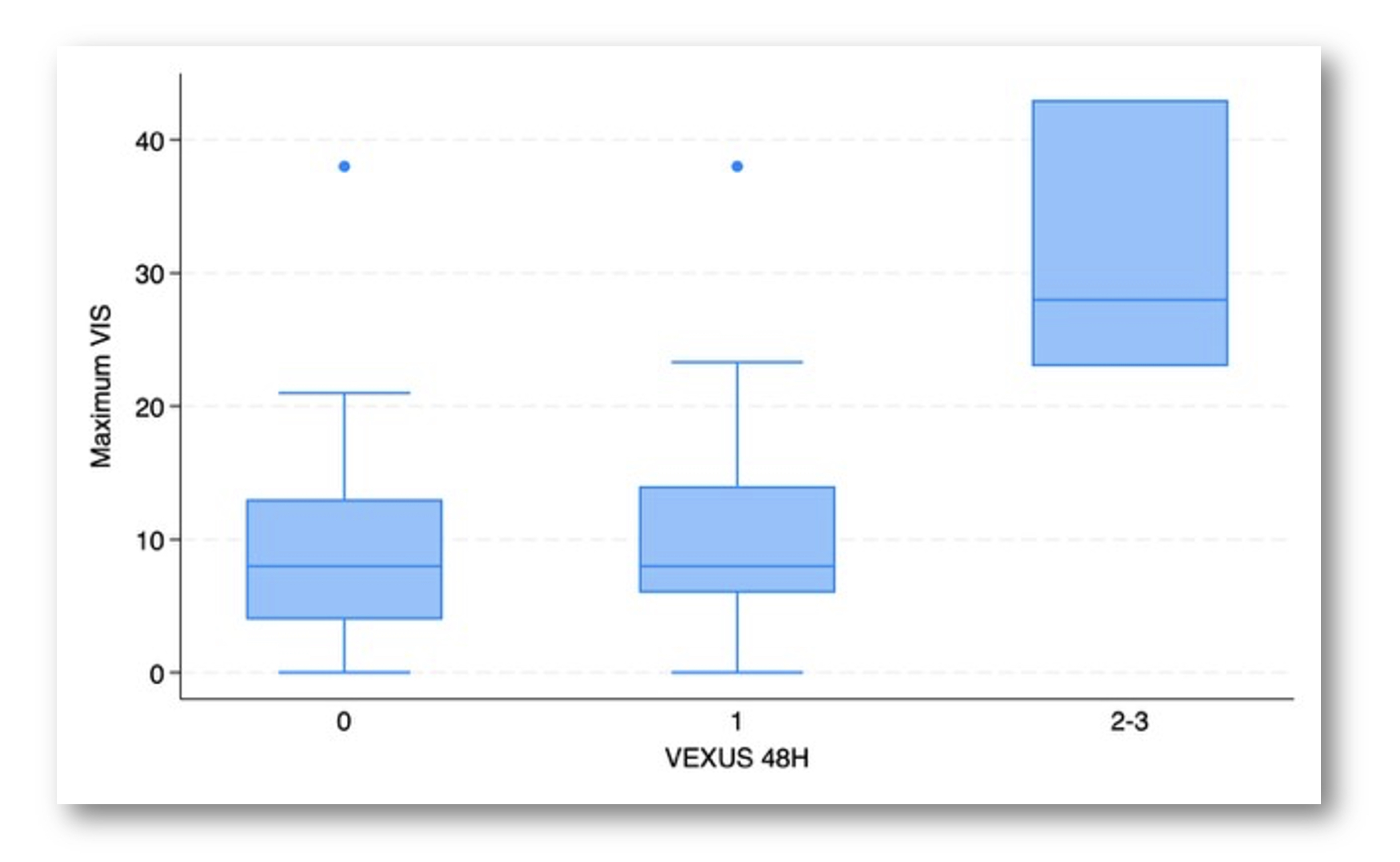

Supplement: Supplementary file 3 — (JPG.456 KB) [file 431_2026_6999_MOESM3_ESM.jpg]

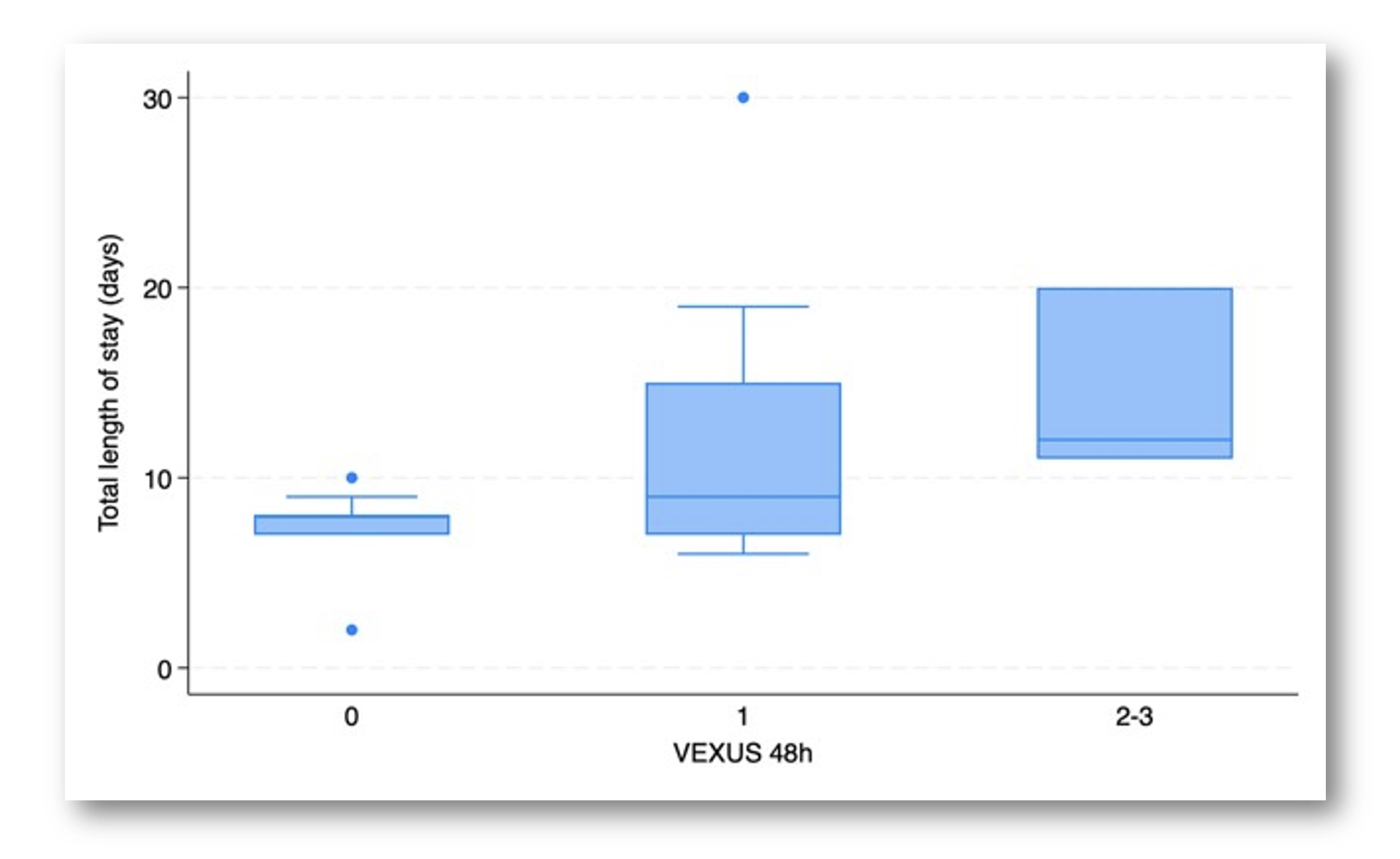

Supplement: Supplementary file 4 — (JPG.386 KB) [file 431_2026_6999_MOESM4_ESM.jpg]

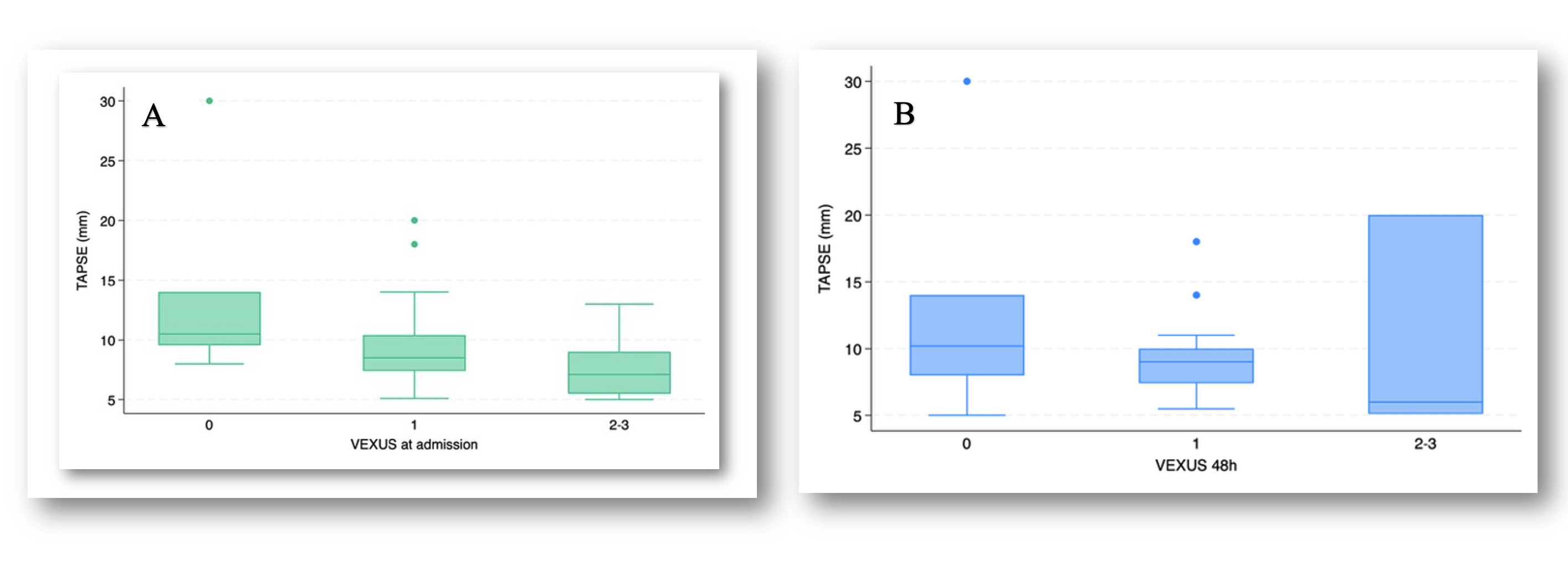

Supplement: Supplementary file 5 — (JPG.394 KB) [file 431_2026_6999_MOESM5_ESM.jpg]
